# Variation of the Polyphenolic Composition and Antioxidant Capacity of Freshly Prepared Pomegranate Leaf Infusions over One-Day Storage

**DOI:** 10.3390/antiox10081187

**Published:** 2021-07-26

**Authors:** Manyou Yu, Irene Gouvinhas, Ana Barros

**Affiliations:** 1 Centre for the Research and Technology of Agro-Environmental and Biological Sciences (CITAB)/Inov4Agro (Institute for Innovation, Capacity Building, and Sustainability of Agri-Food Production), University of Trás-os-Montes and Alto Douro (UTAD), 5000-801 Vila Real, Portugal; igouvinhas@utad.pt (I.G.); abarros@utad.pt (A.B.); 2 Department of Chemistry, School of Life Sciences and Environment, University of Trás-os-Montes and Alto Douro (UTAD), Quinta de Prados, 5000-801 Vila Real, Portugal

**Keywords:** pomegranate leaves, infusion, storage, polyphenolic profile, antioxidant capacity

## Abstract

In recent decades, an intensive search for natural and novel types of antioxidant polyphenolics has been carried out on numerous plant materials. However, the current literature has very little information on their storage stability in the form of freshly prepared infusions. This study aims to characterize the polyphenolic composition and the antioxidant capacity of pomegranate (*Punica granatum* L.) leaf infusions over one-day storage (analyzed at 0, 2, 4, 6, 8, and 24 h). Spectrophotometric evaluation demonstrated that the infusion presented no significant changes in the content of total phenols (131.40–133.47 mg gallic acid g^−1^) and *ortho*-diphenols (239.91–244.25 mg gallic acid g^−1^). The infusion also maintained high stability (over 98% and 82%, respectively) for flavonoids (53.30–55.84 mg rutin g^−1^) and condensed tannins (102.15–124.20 mg epicatechin g^−1^), with stable (>90%) potent antioxidant capacity (1.5–2.2 mmol Trolox g^−1^) throughout 0–24 h storage. The main decrease was observed during 0–2 h storage of flavonoids, 8–24 h storage of tannins, and 0–4 h storage of antioxidant capacity. Chromatographic analysis further revealed that 7 decreased and 11 increased compounds were found within 0–24 h storage. The good stability of the total polyphenolics and antioxidant properties might be related to the complex conversion and activity compensation among these compounds. The findings suggest that pomegranate leaf infusion could be of great interest in the valorization of high added-value by-products and in the application of green and functional alternatives in the food-pharma and nutraceutical industries.

## 1. Introduction

Growing health concerns have produced a dramatic shift in the market demand from synthetic antioxidants to natural products [1]. Accordingly, functional foods derived from medicinal plants have been increasingly generated and marketed, largely due to their benefits regarding well-being and the reduction of disease risk [2]. Among others, plant infusions, as one of the most consumed beverages throughout the world, have been considered to be not only representative of healthcare culture traditionally but also abundant in medicinal components in helping to cure, mitigate, or prevent aging, diabetes, cardio-/cerebro-vascular diseases, etc. [3]. 

Infusions can be referred specifically to immersing plant parts in an amount of hot water, allowed to stand for several minutes, and then filtered for drinking with the purpose of savor, flavor, or medicinal properties [4]. This is much faster and more convenient as compared to other aqueous extractive procedures (e.g., maceration, percolation, and decoction). Even some sophisticated medications in the form of pills, capsules, and ointments have failed to completely replace infusion, as the latter is commonly harmless with few side effects (depending on the plants) [4]. 

Many well-known plants have been commercially used in infusions, such as peppermint and chamomile, due to their rich amount of antioxidants [4]. Conventionally, people drink these plant infusions (also called herbal tea or tisane) quickly (e.g., 1 h) once the infusions are freshly prepared. Many natural phenolics have reactive structures that can degrade to some extent during extraction and storage, especially under the conditions of high temperature or excessive light [5,6,7]. 

Furthermore, plant infusion often focuses on the extension in the shelf-life of plant dry products or packaged infusion drinks/foods for a prolonged time. In contrast, information on the stability of phytochemical and bioactive properties of freshly infused plant materials over one-day storage is not yet readily available. Therefore, it is important to investigate the possible variations of phytochemicals and associated antioxidant activity in plant infusions during 24 h storage, in order to better exploit its potential application.

In this context, pomegranate (*Punica granatum* L.), a popular deciduous shrub in the Lythraceae family, is commonly consumed as fresh fruit and processed juice and is a useful matrix for developing functional foods. Its juice, peel, flower, seed, bark, and roots have been well endowed with a range of healthy phytochemicals, such as polyphenolics and fatty acids, with beneficial qualities, such as anti-cancer, anti-microbial, and organ-protective effects [8,9]. Pomegranate leaves (PLs) have been empirically utilized in folk medicine. Its paste or ointment is used as a cure for arthritis and skin injuries [10]. The decoction or infusion has been used to treat renal colic and treat sore throat, urinary tract infection, and digestive disorders [11]. 

The intake of PL ethanolic extracts as dietary supplements has been preclinically demonstrated to assist in controlling obesity, hyperlipidemia, and hypercholesterolemia [12,13]. Moreover, there are clues that pomegranate leaf infusion (PLI) contains no excitatory and cathartic compositions—found in traditional teas and weight-loss drugs—which suggests high safety without diarrhea, nausea, vomiting, and other adverse reactions [12]. Our previous work also proved that methanolic extracts of PLs presented remarkably higher total phenolic content and antioxidant capacity than those of several well-known herbs, such as sage, rosemary, and peppermint [14]. 

Currently, some putative remedies of PLs can include the inhibition of microbial and inflammation [15,16,17]. Nevertheless, knowledge of the bioactive substances and the biological characteristics of pomegranate leaf infusion (PLI) remains scarce, including knowledge of the possible degradation of the polyphenolic composition and antioxidant capacity during 24 h of storage. 

We hypothesize that a freshly prepared infusion (with local potable water) of PL powder could retain a relatively high level of total polyphenols and antioxidant capacity over one-day storage under natural ambient conditions. This could provide more practical information for the potential applications of PLI and utilize it as a feasible source for food and nutraceutical enrichment. Moreover, PLs are generally agricultural and industrial waste whose applications are beneficial for the valorization of by-products with added values to the economy, environment, and human health. 

Thus, the objectives of the present study are (i) to investigate the variations of polyphenolic composition and antioxidant capacity of PLI over one-day storage (analyzed when stored at 0, 2, 4, 6, 8, and 24 h), determined by spectrophotometric and chromatographic approaches; and (ii) to evaluate the relationship between the polyphenolic constituents and antioxidant properties of PLI during different storage times using the Pearson correlation coefficient (PCC) and principal component analysis (PCA). 

## 2. Materials and Methods

### 2.1. Chemicals and Reagents

Folin–Ciocalteu’s reagent, ferric chloride, methanol, aluminum chloride, sodium nitrite, sodium hydroxide, ammonium sulfate, acetic acid, and methylcellulose (1500 centipoises viscosity at 2%) were acquired from Merck (Merck, Darmstadt, Germany). Sodium carbonate, 2,2′-azino-bis (3-ethylbenzothiazoline-6-sulfonic acid) diammonium cation radical (ABTS^•+^), 2,2-diphenyl-1-picrylhidrazyl radical (DPPH^•^), sodium molybdate, potassium persulfate, (±)-6-hydroxy-2,5,7,8-tetramethylchromone-2-carboxylic acid (Trolox), hydrochloric acid, and 2,4,6-tris(2-pyridyl)-s-triazine (TPTZ) were obtained from Sigma–Aldrich (Sigma–Aldrich, St. Louis, MO, USA). 

Authentic standards of the phenolic compounds used in the chromatographic analysis were purchased from Chem-Lab (Chem-Lab N.V., Zedelgem, Belgium), Sigma–Aldrich (Sigma–Aldrich, St. Louis, MO, USA), and Panreac (Panreac Química S.L.U., Barcelona, Spain), including protocatechuic acid, *p*-hydroxybenzoic acid, benzoic acid, caffeic acid, neochlorogenic acid, chlorogenic acid, vanillic acid, syringic acid, myricetin-3-*O*-glucoside, *p*-coumaric acid, rutin (quercetin-3-rutinoside), ellagic acid, ferulic acid, apigenin-7-*O*-glucoside, rosmarinic acid, luteolin, quercetin, *trans*-cinnamic acid, kaempferol, gallic acid, tyrosol, caftaric acid, catechin, gentisic acid, epicatechin, 4-hydrocinnamic acid, luteolin-7-*O*-glucoside, isorhamnetin-3-*O*-glucoside, oleuropein, resveratrol, and *trans*-stilbene. 

All the chemicals and reagents used in the experiments were of analytical or chromatographic grade. Ultrapure water was obtained using a Water Purification System (Arioso Power, Human Corporation, Seoul, Korea).

### 2.2. Plant Materials

Plant species of *Punica granatum* L. (pomegranate) was botanically authenticated by Dr. João Rocha (Chemistry Centre, Vila Real, UTAD, Portugal). PL samples were hand-picked randomly from a pool (*n* > 10) in June 2019 that were naturally growing in the Botanical Garden of UTAD (GPS: 41.288° N, 7.739° W; Vila Real, Portugal), which belonged to the international network of botanical gardens. Pomegranate was present in the Garden Fruits Collection (more detailed information was available at http://jb.utad.pt/, accessed on 2 June 2021). 

The collected samples were immediately rinsed with potable water, and dried until complete dehydration (40 °C, 72 h) in a Drying Cabinet (LEEC, Nottingham, UK), before being ground into a fine powder with a blender (MB 800, KINEMATICA AG, Malters, Switzerland), and hermetically stored in the dark at room temperature (RT) until analysis. Experimental research and field studies on plants (either cultivated or wild), including the collection of plant material, complied with the relevant institutional, national, and international guidelines and legislation.

### 2.3. Preparation of Plant Infusion

Infusions of PLs were prepared by hot-water extraction. Briefly, around 2 g of dried PLs was soaked in 400 mL of tap water (heated at 80 °C) in a beaker and steeped at room temperature (20–25 °C) for 20 min until cool down. The pH of the local potable water (UTAD, Vila Real, Portugal, 41.288° N, 7.739° W) was approximately at 6.0 as measured by the pH indicator sticks (MACHEREY-NAGEL GmbH &Co. KG, Düren, Germany). After filtration by cotton gauze, the filtrate was secondly filtrated by low ashless qualitative filter paper (FILTROS ANOIA, S.A. Barcelona, Spain) in a measuring cylinder to obtain the final filtration volume (≈330 mL). 

The filtrates were stored in an Erlenmeyer flask (DURAN, Mitterteich, Germany) on the lab table at room temperature (20–25 °C) under natural light overnight. The bottleneck of the flask was superficially covered with silver paper. Subsequently, the filtrates were homogenized and analyzed after stored at 0, 2, 4, 6, 8, and 24 h. The infusions were prepared by extracting PLs in triplicate, and each of the triplicates was repeated three times for each investigated phenolic class at a given storage time (3 × 3).

### 2.4. Determination of Phenolic Content

The contents of total phenols, *ortho*-diphenols, and flavonoids of PLI were determined by spectrophotometric methods according to the previous description [18]. The content of tannins was evaluated by the methylcellulose (MC) methodology as reported by Dambergs, et al. [19] with some modifications.

For the evaluation of total phenol content, diluted infusion (20 μL), 10% (*v:v*) Folin–Ciocalteu reagent (100 μL), and 7.5% (*w:v*) sodium carbonate (80 μL) were mixed in sequence. The mixture was incubated for 30 min at 42 °C in the dark and measured at 750 nm using gallic acid as the standard. The results are expressed in milligrams of gallic acid per gram of plant dry weight (mg GA g^−1^ DW). For the assessment of *ortho*-diphenols content, 5% (*w:v*) sodium molybdate (40 μL) was added to the diluted infusion (160 μL). The mixture was left for 15 min at RT, protected from light, before the read of absorbance at 375 nm. The results are expressed in mg GA g^−1^ DW. 

For the quantification of the flavonoid content, diluted infusion (24 μL) and 5% (*w:v*) sodium nitrite (28 μL) were mixed. After 5 min at RT, 10% (*w:v*) aluminum chloride (28 μL) was added to the mixture and reacted for 6 min. Then, 1 M sodium hydroxide (120 μL) was added, and the absorbance of the mixture was measured at 520 nm after agitation for 30 s in a microplate reader. The results are shown as milligrams of rutin per gram of plant dry weight (mg RU g^−1^ DW). The aforementioned assays were undertaken with a microplate reader (Multiskan FC Microplate Photometer, Thermo Fisher Scientific, Vantaa, Finland) in 96-well microplates (PrimeSurface MS-9096MZ, Frilabo, Maia, Portugal) with a final volume of 200 µL.

The content of condensed tannins was evaluated both in the treatment and control groups simultaneously by adding MC (600 μL) as treatment or ultrapure water as a control to the infusion (200 μL) in a 2 mL Eppendorf. The mixture was stirred manually for 3 min at RT. Saturated ammonium sulfate (400 μL) and water (800 μL) were added successively both in the treatment and control groups until 2 mL of total volume was reached. 

The final mixture was vortexed and kept for 10 min. After centrifugation (10,000 rpm, 16 °C, 5 min), the absorbance was read at 280 nm, by using a conventional spectrophotometer (Helios Gamma UV Spectrophotometer, Thermo Electron Corporation, Warwickshire, UK). The absorbance of tannins was obtained by subtracting the treatment absorbance from the value registered from the control using epicatechin as the standard. The results are described in milligrams of epicatechin per gram of plant dry weight (mg EC g^−1^ DW).

### 2.5. Evaluation of In Vitro Antioxidant Capacity

The antioxidant capacity of PLI was determined by ABTS, DPPH, and FRAP (ferric reducing antioxidant power) spectrophotometric methods, as reported by Mena, et al. [20] with some adjustment. ABTS working solution (188 μL) and sample dilutions (12 μL) were mixed and reacted for 30 min at RT. Ultrapure water was used as a blank. The absorbance was read at 734 nm. A mixture of DPPH working solution (190 μL) and sample dilutions (10 μL) was stood for 15 min at RT. We read the absorbance at 520 nm with hydro-methanol used as the blank. 

The FRAP working solution was prepared by mixing 10-volume acetate buffer (300 mM, pH = 3.6), 1-volume TPTZ (10 mM dissolved in hydrochloric acid), and 1-volume ferric chloride (20 mM in water). The mixture was maintained at 37 °C for 10 min before use. The reaction of FRAP working solution (180 μL) and sample dilutions (20 μL) was kept at 37 °C for 30 min with the absorbance read at 593 nm. The three antioxidant assays were adapted to microscale using 96-well microplates (PrimeSurface MS-9096MZ, Frilabo, Maia, Portugal) and microplate readers (Multisskan GO Microplate Photometer, Thermo Fisher Scientific, Vantaa, Finland), using Trolox as standard. All the results are expressed in mmol Trolox per gram of plant dry weight (mmol Trolox g^−1^ DW).

### 2.6. Chromatographic Analysis of Phenolic Compounds

Reverse Phase–High Performance Liquid Chromatography–Diode Array Detector (RP-HPLC-DAD) system (Thermo Finnigan, San Diego, CA, USA) was carried out to determine the polyphenolic profile of each plant extract, as previously described [18]. The analysis equipment was composed of three parts, including an LC pump (Surveyor), autosampler (Surveyor), and PDA detector (Surveyor). Triplicates of infusions analyzed at different storage times and authentic standards were prepared and filtered through 0.45 μm PVDF filters (Merck Millipore, Bedford, MA, USA) and injected into an ACE C18-HL (Hi-Load) HPLC Column (250 mm × 4.6 mm, 5 μm particle size; ACE, Aberdeen, Scotland), using a mobile phase composed of water/formic acid (99.9:0.1, *v:v*) (solvent A) and acetonitrile/formic acid (99.9:0.1, *v:v*) (solvent B). 

The linear gradient program (t = min–%B) was: t = 0–0%; t = 5–0%; t = 20–20%; t = 35–50%; t = 40–100%; t = 45–0%; and t = 65–0%. The injection volume was 20 μL, and the flow rate was kept at 1.0 mL min^−1^. UV/Vis detection was recorded from the 200 to 600 nm range. Peaks were monitored at 280 and 330 nm, and identified by congruent retention times compared with standards and the reference literature. Data acquisition, peak integration, and analysis were performed using Chromeleon software (Version 7.1; Thermo Scientific, Dionex, Sunnyvale, CA, USA). Concentrations of the identified compounds were expressed in milligrams per ten grams of plant dry weight (mg 10 g^−1^ DW).

### 2.7. Data and Statistical Analysis

All the measurements of polyphenolic composition (spectrophotometric and chromatographic methods) and antioxidant capacity (ABTS, DPPH, and FRAP assays) of the plant infusions at different storage times were performed in triplicate with the results presented as the mean ± standard deviation (SD, *n* = 3). The obtained data were subjected to analysis of variance (ANOVA) and a multiple range test (Tukey’s test) using IBM SPSS statistics 21.0 software (SPSS Inc., Chicago, IL, USA). Pearson correlation coefficient (*r*) was carried out to establish correlations between polyphenolic composition and antioxidant capacity. Principal component analysis (PCA) was conducted using a multivariate matrix of the set of measured parameters, statistically determining the similarities and differences between these factors via XLXTAT software version 2017.03 (Addinsoft SARL, Paris, France).

## 3. Results and Discussion

### 3.1. Effect of Storage Time on the Polyphenolic Composition of PLI during One-Day Storage

#### 3.1.1. Change in the Levels of Total Phenols (TPs), *Ortho*-Diphenols (OPs), Flavonoids (TFs), and Condensed Tannins (CTs) 

The infusion extraction was applied to obtain the bioactive compounds. This aqueous-based procedure was the cheap, simple, scalable, and non-toxic to perform, common to extract tannins with residues friendly to the environment, and relevant to human bioavailability [21]. The chemical contents of PLI are shown in Table 1. The content of TFs (53.30–55.84 mg RU g^−1^ DW) and CTs (102.15–124.20 mg EC g^−1^ DW) significantly impaired within 24 hours (*p* < 0.001), whereas no notable difference was found during 2–8 h of storage (*p* > 0.05). Moreover, there was no linear change trend in the content of TPs (131.40–133.47 mg GA g^−1^ DW) and OPs (239.91–244.25 mg GA g^−1^ DW). The highest amounts of different chemical classes were all recorded at 0 h storage, with a range of decreasing percentage from 1.55% to 17.75%.

**Figure 1:**
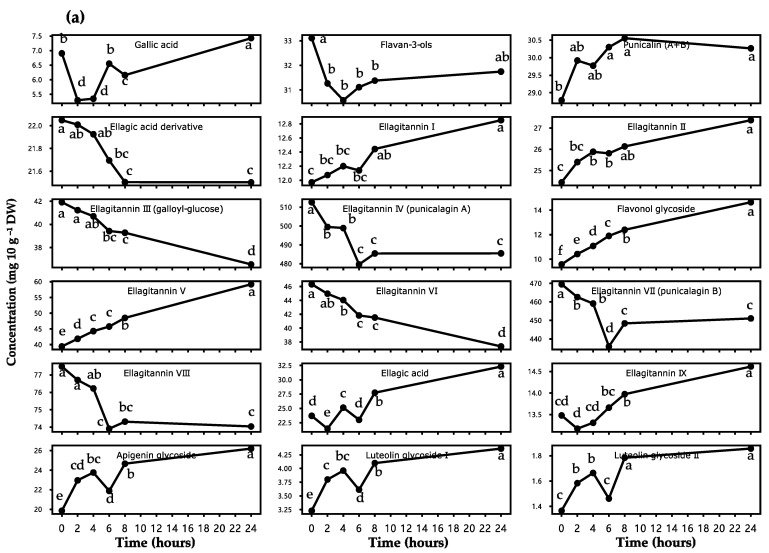

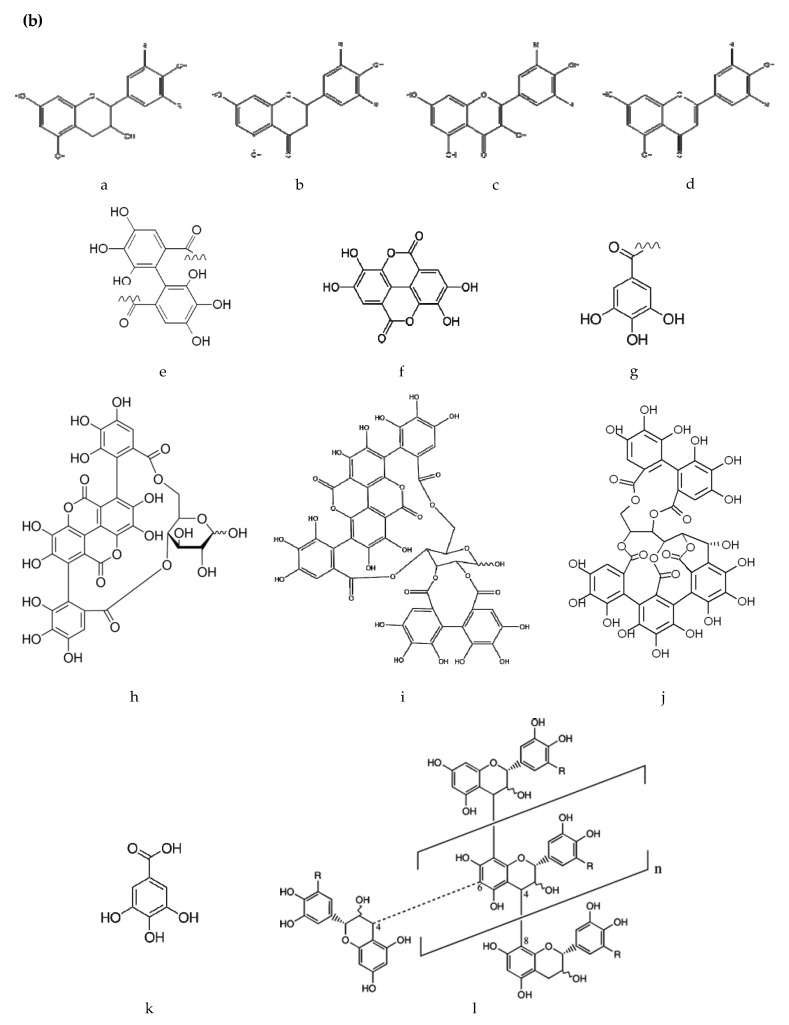
(**a**) Changes of individual compounds identified by RP-HPLC-DAD analysis. Concentrations of individual identified compounds are expressed as milligram per 10 grams of plant dry weight. Means (*n* = 3), showed as blank dots in each sub-figure, followed by different lowercase letters that report significant differences (*p* < 0.05) of phenolic concentrations during 0–24 h storage, according to Tukey’s test. (**b**) Structures of polyphenolic compounds present in the pomegranate leaf infusions. a: flavan-3-ols; b: flavanone; c: flavonol; and d: flavone. Basic structures of ellagitannins, such as e: HHDP acid, f: ellagic acid, g: galloyl group; h: punicalin; i: punicalagin; j: other possible ellagitannin (e.g., castalagin); k: gallic acid; and l: condensed tannin.

The results showed that the content of TFs and CTs significantly decreased during 0–2 h storage after the PLI was freshly prepared (Table 1). One possible cause was due to decreased catechins content, as shown in Figure 1a, which could be associated with the higher initial temperature and oxygen concentration. The declining trend of catechins with rising temperature and oxygen can play an important role in the color alteration and antioxidative degradation of tea [22]. CTs (also known as proanthocyanidins, PAs) are polymers formed by monomeric flavan-3-ols units and connected via C–C or C–O–C bonds [6]. This reactive construction can lead to an oxidative cleavage or hydrolysis of the chemical structures [23], which may explain the observed significant reduction of CTs before 2 h storage (Table 1). 

Our results revealed that the content of TFs and CTs maintained great stability within 2–8 h storage (Table 1). Catechins can undergo epimerization, such as the epigallocatechin gallate (EGCG) that can be easily transformed to the stable gallocatechin gallate [22]. Additionally, studies suggested PAs are either hydrolyzed or polymerized during cooking and processing due to the conformational flexibility of tannins, thus, becoming difficult to separately extract or quantify [6]. Thus, some tannins could be de-polymerized into small tannins or flavonoids, while some others could be re-polymerized into larger tannins [23]. 

Therefore, the products generated from the hydrolysis or oxidative decomposition of CTs likely enhanced the TFs content during 2–8 h storage. Meanwhile, this also potentially explained the lower degradation percentage of TFs (4.54%) compared to that of CTs (17.75%) (Table 1) indicating that some tannins were possibly decomposed into flavonoids. Furthermore, after 2 h storage along with the cooling down of PLI, the stable room temperature also helped to stabilize the content of TFs and CTs.

As shown in Table 1, a significant decrease in the content of TFs and CTs was found between 8–24 h storage. With the extension of storage at ambient conditions, the pH of PLI may decrease resulting from the carbon dioxide existing in the environment. Both flavonoids and their glycosides had been reported to endure polymerization under more acidic pH situations and then to form non-phenolic pigments, which causes the browning and deterioration of tea [24]. 

Moreover, tannins produced by either the hydrolysis or the re-polymerization of original tannins in the PLI were assumed to undergo stronger oxidative destruction throughout 8–24 h storage, consequently causing a higher decrease of CTs in this period [6,23]. However, due to the complicated structures and the conformational flexibility of tannins aforementioned, it was difficult to clarify the main triggers (e.g., the temperature, oxygen, pH, light, and microbes) or clear pathways of the change of tannins. Elaborate experiments and advanced techniques need to be further performed.

Evidently, hydrolyzable tannins (HTs), another primary category of tannins has been also been proven to be abundant in PL extracts [16,25,26]. Both CTs and HTs in plants have reactive hydroxyl groups, thus, imparting free radical inhibitory activity to tannins. Apart from CTs, HTs are also capable of retaining their original structure over the drying process and experiencing hydrolysis, decomposition, and polymerization during hot-water extraction [6]. 

High-molecular weight HTs (e.g., ellagitannin oligomers) can be degraded into monomers (e.g. punicalagin and punicalin), intermediate-molecular weight fragments (e.g., HHDP (3, 4, 5, 3′, 4′, 5′—hexahydroxydiphenic acid) glucoside, and PGG (pentagalloylglucose)), phenolic acids (e.g., ellagic acid and gallic acid) [27,28]. These degraded compounds also belonged to the bioactive polyphenolics. HHDP and PGG moieties can be spontaneously converted to other HTs by rearrangement or intermolecular C-C coupling [21]. Furthermore, as the degree of tannins polymerization increased, the number of hydroxyl groups became higher. In addition, some polyphenols, such as lignans, coumarins, or stilbenes, may exist in the PLI, awaiting identification [29]. 

Thus, in short, we supposed that the stability in the content of TPs and OPs within 24 h storage, as well as the balance in the quantity of TFs and CTs during 2–8 h storage, could arise from the structural diversities and reactive characteristics of polyphenolics present in PLI, namely by the epimerization of catechins and by the hydrolysis, polymerization, and depolymerization of tannins, among others. However, the decline of TFs and CTs content between 0–2 h and 8–24 h of storage was closely associated with the oxidation of catechins, polymerization of flavonols or flavones, and oxidative destruction of hydroxyl groups, possibly with the impact of temperature, illumination, and pH.

#### 3.1.2. Change in the Polyphenolic Profiles

To obtain a closer insight into the polyphenolic profiles and their changing features of the PLI during one-day storage, twenty individual phenolics were identified by the authentic standards resorting to RP-HPLC-DAD and with comparison to the literature by the retention time, UV/Visible *λ*_max,_ and spectra (Figure S1, Table S1, and Table 2). Eighteen of the identified compounds were quantified with calibration curves built by the standards (Figure 1a). Generally, the most widespread compounds of PLI identified in this work were ellagitannins (ETs) followed by flavonoid glycosides and phenolic acids. A significant change (*p* < 0.05) in the concentration of individual polyphenolics of PLI was observed over 24 h of storage.

The results revealed that a significant decrease of the phenolic constituents throughout 24 h storage occurred in flavan-3-ols (decrease of 4.11%, FO), ellagic acid derivatives (2.48%, EAD), ellagitannin-III (12.82%, ET-III), ellagitannin-IV (5.29%, ET-IV), ellagitannin-VI (19.36%, ET-VI), ellagitannin-VII (3.93, ET-VII), and ellagitannin-VIII (4.45%, ET-VIII), as shown in Figure 1a and Table 2. 

(i) Part of EAD degraded to ellagic acid by hydrolysis [30], leading to a decrease during storage. 

(ii) The content of FO declined faster in the first two hours, remaining at practically constant levels afterward. In the initial storage time, the temperature of the PLI was higher than the RT, which can affect the stability of FO. These FO (also called catechins) can be further assumed as epigallocatechin gallate or epigallocatechin since they had three hydroxyl groups and were, thus, more reactive to degradation than other types of catechins. When stored from 2 to 24 h, the FO content showed no significant change, likely due to the stable temperature (equal to RT) and acidic condition, consistent with a previous report [22]. 

(iii) ET-IV and ET-VII had lower degradation percentages over the day and presented the highest concentrations at 0 h storage (512.65 mg/10 g DW and 469.48 mg/10 g DW, respectively), followed by ET-VIII (77.48 469.48 mg/10 g DW). 

However, ET-III and ET-VI presented the lowest amounts at 0 h and higher deterioration at 24 h. The stability of ET-III, ET-IV, ET-VI, and ET-VIII was more perceptible in the first 4 h and secondly stable at 6–8 h. Except for other degraded phenolics, the peak of ET-VII was found at 6 h storage and not at 0 h, which was speculated as HHDP-glucosides, the intermediate molecular weight fragments. The concentration of ET-VII increased from the partial breakdown of polyphenols (high-molecular weight ETs) before 6 h and then decreased by subsequent chain destruction into low-molecular compounds, such as ellagic acid [30,31,32].

On the other hand, the results showed that eleven phenolics increased significantly during one-day storage (Figure 1a and Table 2), as follows. 

(i) The rising contents of gallic acid (increase of 7.59%, GA) and ellagic acid (36.39%, EA) were obtained from the HTs, which consisted of ester of GA in gallotannins and EA in ETs [6,27]. The higher increasing percentage of EA implies that more ETs and more extensive hydrolysis of ETs occurred in the storage of PLI. 

(ii) The amount of apigenin glycoside (31.98%, AG), luteolin glycoside I (35.9%, LG-I), and luteolin glycoside II (36.03%, LG-II) raised during 0–4 h and 6–8 h of storage, mostly coming from polymerization of flavonoids; whereas a reduction between 4 h to 6 h was observable likely due to hydrolysis of these polymers, partly conducive to the stability of TFs content during this storage period [6,24]. The ascent of flavonol glycoside (52.98%, FG) was continuous and comparatively higher within 24 h, possibly owing to more hydroxyl groups present in flavonols than that in flavones (apigenin and luteolin) [6]. 

(iii) The concentration of ellagitannin V (ET-V) grew the most (50.08%) among the increased tannins, followed by ellagitannin II (11.99%, ET-II), ellagitannin IX (8.41%, ET-IX), ellagitannin I (7.37, ET-I), and punicalin (5.14%). It was noted that ETs were complex monomeric or oligomeric polyester molecules composed of HHDP and a related sugar or polyol [27,33]. They can readily undergo manifold chemical reactions, such as transformation, isomerization, and oligomerization, which determine the important structural diversity of ETs [27,33]. 

Moreover, ETs can be readily hydrolyzed in acidic or basic solutions, where the ester bonds in the polymer were cleaved during hydrolysis [27]. Thus, it can be assumed that some complicated convertible actions happened during the storage of PLI. Additionally, Ito, et al. [28] isolated two new oligomeric ETs, three known oligomeric ETs, and three known monomeric ETs from the arils and pericarps of pomegranate, all of which exhibited potent inhibitory capacity towards the formation of advanced glycation end products. 

Elucidating the native structural variations of ETs was often a challenge, yet, according to other relevant reports [16,27,28,30,33,34], ET-I, -II, -V, and -IX can be assumed as ellagitannin monomers or HHDP intermediates, of which the ascending concentrations were likely attributed to the decomposition of some uncertainly identified ellagitannin oligomers or monomers or to the intricate transformation of other ETs or gallotannins.

The polyphenolic profiles of PLs extracted by using organic solvents, such as methanol, ethanol, acetone, hexane, petroleum ether, ethyl acetate, etc. have been extensively researched previously [15,35,36]. In the last decade, some studies reported the phytochemical screening and medicinal properties of the aqueous PL extracts mostly using laboratory water, such as distilled, deionized, or ultrapure grade water [15,25,37,38,39]; however, only some investigated the specific constituents. Orgil, et al. [40] studied fresh PLs by extraction with cold distilled water and quantified the content of four bioactive compounds, including punicalagin, gallagic acid, ellagic acid, and gallic acid. 

Uysal, et al. [37] found that chlorogenic acid was the most abundant component in the decoction of dry PLs, followed by gallic acid, *p*-coumaric acid, ferulic acid, and rutin. Recently, by means of LC-ESi-MS, more phytochemicals were tentatively identified from the leaf infusion (immersed at 90–95 °C) of Palestinian pomegranates—mainly quinic acid, corilagin, granatin B, brevifolin carboxylic acid, and eschweilenol C [38]. 

However, many ingredients of aqueous PL extracts still have not been confirmed thus far, possibly as a result of different regions, varieties, plantation, or growth stages referred to plant collection. In our study, the PLI was featured with the simplest preparation (soaked in boiled tap water) and the most common storage (exposed in natural ambient conditions), consistent with human dietary habits. Our results enabled an update of the incomplete information of PLI with potentially high availability in food and nutraceutical industries.

**Figure 2:**
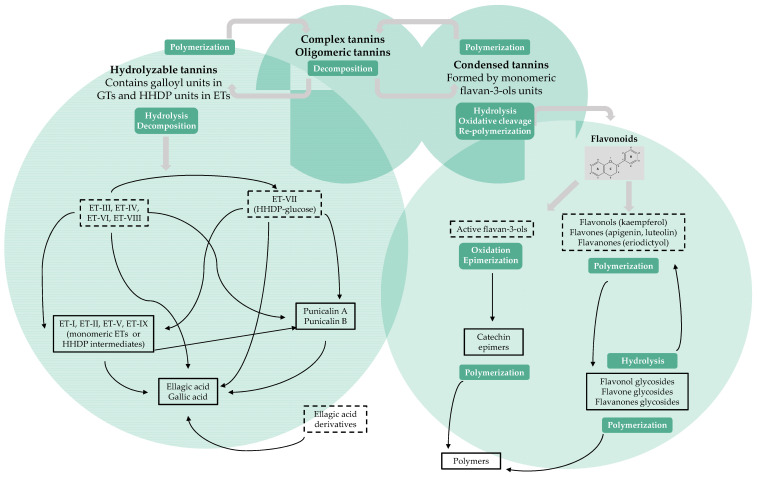
The hypothesized transformation action of the determined polyphenolic composition and the putative compounds of pomegranate leaf infusion during one-day storage. The relevant compounds were classified into hydrolyzable tannins (HTs), condensed tannins (CTs), flavonoids, and complex or oligomeric tannins. Polyphenolic compounds enclosed in dashed (or solid) boxes indicated decreased (or increased) concentrations. Reactions enclosed in green rounded rectangles indicated the possible transformation mechanisms between the compounds.

Apart from phenolic profiling, an outline regarding the hypothetical synergistic or antagonistic transformation of identified polyphenolics of PLI during one-day storage was roughly envisaged for the first time (Figure 2). In keeping with the literature references [6,7,22,23,27,30,31,32,33,41,42,43,44], this transformation effect of these polyphenolic compounds might involve their hydrolysis, polymerization, antioxidative cleavage, and conformational changes, among others, presumably linking to environmental factors, such as temperature, oxygen, pH, and light. 

This picture could be useful to better understand the stability of total phenolic content and the change of individual phenolics of PLI during one-day storage—particularly ETs—and, accordingly, improve the use efficiency. The benefits of ETs-rich foods are promising and deserve to be part of a healthy diet as functional foods [27]. However, ETs from different food sources present diverse hydrolysis pathways, generating distinct products, which arise from the large structural variety of these compounds. Some hydrolyzable routes and products of food ETs had been well-described, such as the fruit of pomegranate [34] and *Rubus* genus [31]. By contrast, our results of the polyphenolic transformation of PLI require further illustration. 

### 3.2. Effect of Storage Time on the Antioxidant Activity (AOA) of PLI during One-Day Storage 

The AOA of PLI was determined using three different assays, namely the ABTS and DPPH free radical scavenging capacity and the ferric reducing antioxidant power (FRAP) since no single assessment was enough to evaluate AOA, and these in vitro screening methods were low-cost and high-throughput tools used to discover potential antioxidant sources [45,46]. As shown in Table 1, significant changes (*p* < 0.01) in the value of ABTS, DPPH, and FRAP were investigated in the PLI during one-day storage at RT. However, it can be seen that the main decrease in the three AOA assays was observable before 4 h of storage. 

Importantly, the results in this study revealed that PLI exhibited great AOA in the overall storage ranging from 1.5 to 2.2 mmol Trolox g^−1^ DW and also maintained at high levels of AOA, more than 90% after 24 h, which hinted the promising bioactive stability of the PLI. Uysal, et al. [37] described that both methanolic and aqueous extracts of PLs possessed higher antioxidant activity as compared to other leaves from avocado, walnut, mulberry, fig, carob, lemon, grape, and loquat, all of which are commercially widespread and pharmacologically applicable throughout the world. 

Moreover, comparing with young leaves of twenty edible and medicinal plants, the ethyl acetate and ethanol extract of PLs displayed the superior inhibition towards DPPH radical and glycation, which may be related to its high amounts of total phenolics and flavonoids [47]. In addition, the AOA of pomegranate juice as well as its high ratio of punicalagin and pedunculagin was three-times higher than that of green tea by using ABTS, DPPH, and FRAP methods [48]. 

PLs were assessed to have comparable or even higher levels of phytochemicals and AOA than other parts of pomegranate, such as the fruit juice, flower, stem, peel, and seeds, depending on the conditions of maturity, variety, climate change, and geographic location among others [32,38,49,50]. 

The results showed that no significant difference in the ABTS, DPPH, or FRAP assay was detected during 2–24 h of storage, during 2–4 h and 6–24 h of storage, or during 0–2 h and 8–24 h of storage, respectively. In principle, the AOA of phenolic compounds is to donate the hydrogen atom (H) or electron to reduce metal ions, carbonyls, and radicals, thereby, preventing and treating the oxidation of lipids, proteins, and DNA [51]. 

In the storage of PLI, the quantitative and structural alterations of different phenolic compounds might occur, thus, affecting the reactions between these antioxidants and radicals or ferric irons and, consequently, causing the difference of evolution in the level of different AOA assays. In addition, the results of these methods might vary to a degree, even for the same infusion samples, since the reaction was also largely dependent on the oxidizing agent prepared to generate the radicles [51]. 

However, by the three methods, AOA of PLI displayed significant stability after stored for 4 h, which was likely associated with several polyphenolic complexes present in PLs and with the modification of their phenolic profiles, particularly CTs and HTs (Figure 2). This was the first work describing the degradable evolution of AOA concerning the one-day storage of PLI. Nevertheless, our results are in agreement with studies in the literature that researched pomegranate fruit and highlighted that the loss of initial phenolics could be compensated by newly produced phenolics with equal or improved AOA [41]. 

For instance, the compounds, like punicalin, gallagic acid, gallic acid, and ellagic acid, which were hydrolyzed from punicalagin, also possessed high AOA [34]. Therefore, the conversion of phenolics and compensation of activity could greatly explain the antioxidant stability of PLI during 4–24 h of storage.

With the extension of storage time, the level of pH, light, temperature, and oxygen could change, which could influence the polyphenolic structure and profile of the PLI. The high retention percentage (>90%) of AOA after one-day storage of the PLI was underlined in this work presumably as a result of the synergistic actions of tannins in terms of their manifold structures. The AOA of tannins in foods and beverages was first demonstrated when they were found to restraint the oxidation of ascorbic acid [33]. 

Tannin-rich foods have been widely reported to possess potential antioxidant, anti-cancer, and organ-protective effects [6]. Krook, et al. [44] evaluated the chemical stability of two tannins (belong to HTs and CTs, respectively) in aqueous solutions under natural and laboratory soil conditions, which had different half-lives at elevated temperatures. It was pointed out that tannins rapidly decomposed at biological pH (7.4) unless oxygen was excluded from the system. 

The same authors [44] also suggested tannins were relatively stable at RT in slightly acidic soils but both higher temperature and neutral to basic pH may promote chemical degradation. Structurally, the number, structure, and location of the hydroxyl group at the aromatic ring have a profound effect on the AOA. The neighboring substituents of the phenolic hydroxyl group may reinforce conjugation, thus, contributing to their outstanding AOA of tannins [33]. 

Moreover, the free radical scavenging properties of galloylated catechins are stronger than nongalloylated catechins. Cai, et al. [52] and Niaz and Khan [6] indicated that the number of polyphenolic galloyl moieties in tannins had a direct relation to higher protein-binding and anticancer efficacy. It was suggested that the presence of the galloyl or hydroxyl group at the 3′- or 5′-position in the B-ring plays the most important role in their free radical scavenging capacity [33]. Furthermore, the higher the degree of polymerization of tannins, the more the number of hydroxyl groups increased, triggering more radicals scavenged per molecule [6]. These reports are consistent with our results as shown in Figure 1b. 

### 3.3. Correlation Analysis

#### 3.3.1. Pearson Correlation Coefficient (PCC)

The PCC is usually applied to express the strength between two continuous variables, which is useful to demonstrate how the response variables are related mathematically and to understand the proportion of the fluctuation of one variable that was predictable from the other variable. We also analyzed the statistical significance of each Pearson’s *r*, which can be achieved by calculating the *p*-value. 

As shown in Table 3, the *r* value of PCC demonstrated a positive significant correlation of TFs and CTs with radical scavenging and ferric reducing activities orderly measured by ABTS (*r* = 0.98, *r* = 0.99), DPPH (*r* = 0.89, *r* = 0.83), and FRAP (*r* = 0.94, *r* = 0.86), respectively, indicating that the evolution of these phenolic compounds significantly contributed to the good antioxidant stability of PLI during one-day storage. This observation was strengthened by several studies [17,41] reporting that the antioxidant and anti-diabetic activity of PL extracts was linked to the presence of flavonoids and tannins. 

In addition, some researchers stated their results in favor of a positive correlation between AOA and TPs content [41,53]. However, other authors found that the decreased TPs content did not agree with the loss of AOA, largely due to the formation of degradation products of polyphenols [43,54]. In reality, no significant correlation of AOA with either TPs or OPs was found through our work, which could be explained by the existence of polyphenolic transformation and the modification of polyphenolic concentration during storage—consistent with Moser, et al. [43] and Rocha-Parra, et al. [54].

On the other hand, the decreased polyphenolics, except catechins, were significantly correlated with the degradation of AOA (Table 3). Separately, EAD, ET-III, ET-IV, ET-VI, and ET-VIII were found to highly correlate with ABTS scavenging capacity (*r* = 0.84, *r* = 0.84, *r* = 0.91, *r* = 0.86, and *r* = 0.89, respectively) and with ferric reducing power (*r* = 0.91, *r* = 0.82, *r* = 0.88, *r* = 0.85, and *r* = 0.92, respectively). In addition, the DPPH scavenging activity was positively correlated with the decreasing of ET-IV, ET-VII, and ET-VIII (*r* = 0.95, *r* = 0.94, and *r* = 0.89, respectively). 

Furthermore, the results also showed that the evolution of ET-III, ET-IV, ET-VI, and ET-VIII was significantly correlated with the content of TFs (*r* = 0.89, *r* = 0.95, *r* = 0.91, and *r* = 0.96, respectively) and CTs (*r* = 0.81, *r* = 0.87, *r* = 0.84, *r* = 0.84, respectively). EAD and ET-VII as well had a significant correlation with the change of TFs (*r* = 0.90 and *r* = 0.84, respectively). However, no association of individual polyphenolic concentration with the evolution of TPs and OPs was recorded. 

Certain highly positive or negative correlations were noted between individual compounds, representing a similar or reverse trend of their evolution. For instance, ET-I was positively linked with ET-II (*r* = 0.94), FG (*r* = 0.96), and ET-V (*r* = 0.98), while negatively linked to ET-III (*r* = −0.95) and ET-VI (*r* = −0.94). These findings can reinforce the view of the presence of some inter-relationship between compounds during storage. 

As we know, the endogenous antioxidant systems in humans is damaged by aging and external factors, such as smoking, drug, alcohol, or poor diet, causing cancer, diabetes, and cardiovascular diseases. Polyphenols originating from medicinal plants have been considered as potent antioxidant agents in the fight against many deleterious and chronic disorders [1,4]. 

Recent studies already demonstrated that PL extracts possessed superior AOA, owing to the various flavonoids and tannins, among others, which are associated with many biological properties, including anti-inflammation, anti-microbial, anti-diabetes, anti-virus, anti-diarrheal, anti-ulcer, and hepato- and nephron- protection [11,13,16,25,26,55,56,57]. Nonetheless, only a few works have studied the relationship between the health-promoting effects and a certain compound isolated from PL extracts; there were scarcely any articles about PLI that illustrated the changing features between AOA and polyphenolic composition. 

Pinheiro, et al. [16] uncovered for the first time that galloyl-HHDP-glucose (the main structure present in ETs) separated from the hydroalcoholic extract and ethyl acetate fraction of PLs ameliorated lipopolysaccharide-induced acute lung injury and attenuated weight loss in mice. In our present study, we first outlined the PLI in regard to its association of the stability of AOA with the change of polyphenolic profiles over one-day storage. However, the potential of individual compounds, their alteration mechanism, and their inter-synergistic or inter-antagonistic action need to be explored with the view to exerting their larger functional profiles as natural substances in the food-pharma and nutraceutical industries.

#### 3.3.2. Principal Component Analysis (PCA)

PCA is one of the most welcome multivariate statistical tools used to explain differentiation between samples and to obtain multiple information on the variables that mostly affect the sample similarities and differences. It involves a mathematical procedure that reduces the dimensionality, produces the dominant patterns in the data matrix in accordance with a complementary set of scores and loading plots, and encompasses the cumulative effects of all critical parameters along with their interaction effects. 

The principal components (PCs) are a linear combination of the original variables that can suggest us how many components are necessary to explain the greater part of variance with a minimum loss of information. For this purpose, we performed PCA on the determined variables of PLI over one-day storage, in order to confirm any relationship between the variables and to emphasize similarities and differences among the different storage times. 

**Figure 3:**
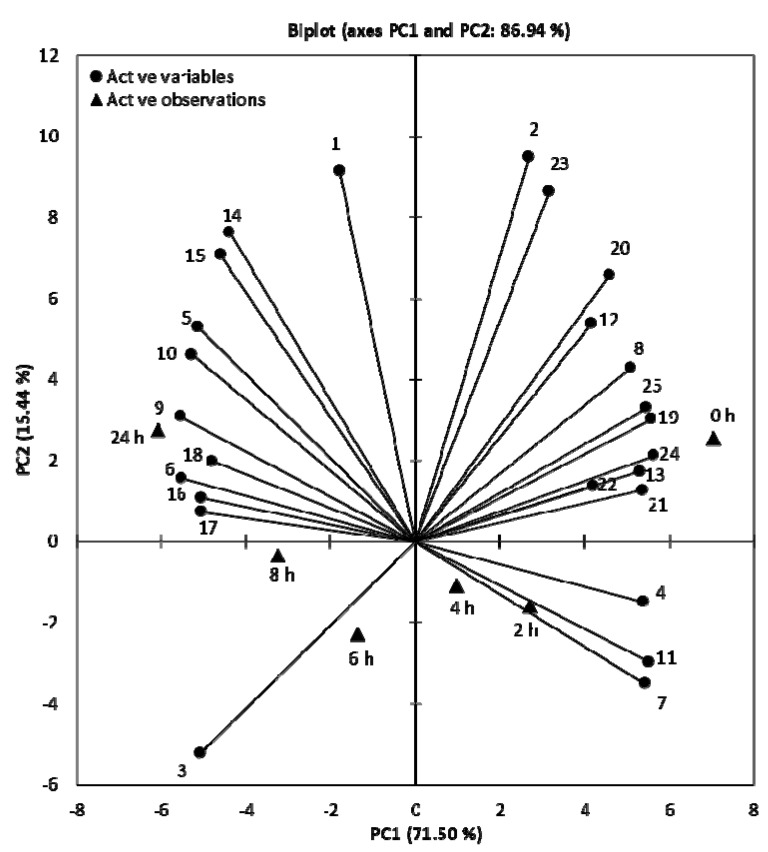
Bi-plot of the PCA analysis performed on the determined 25 variables of pomegranate leaf infusion over one-day storage. Loading plots (vectors representing active variables) and scores plots (triangles representing active observations-different storage times) of PC1 and PC2. Numbers of vectors represented: (1) gallic acid, (2) flavan-3-ols, (3) punicalin, (4) ellagic acid derivative, (5) ellagitannin I, (6) ellagitannin II, (7) ellagitannin III, (8) ellagitannin IV, (9) flavonol glycoside, (10) ellagitannin V, (11) ellagitannin VI, (12) ellagitannin VII, (13) ellagitannin VIII, (14) ellagic acid, (15) ellagitannin IX, (16) apigenin glycoside, (17) luteolin glycoside I, (18) luteolin glycoside II, (19) ABTS, (20) DPPH, (21) FRAP, (22) total phenols, (23) *ortho*-diphenols, (24) flavonoids, and (25) tannins.

The contents of phenolic classes (including TPs, OPs, TFs, and CTs), antioxidant capacities (estimated by ABTS, DPPH, and FRAP), and the concentrations of eighteen polyphenolics identified by HPLC from the different storage times (0, 2, 4, 6, 8, and 24 h) of PLI were subjected to PCA. The statistical analysis retained the first two principal components of which the first (PC1) accounted for 71.50% of the variance, while the second (PC2) explained 15.44% (Figure 3), accounting for a total variance of 86.94%. 

The loading plots (shown as vectors in Figure 3) represented the contributors to the principal components. The levels of antioxidant properties, the contents of phenolic classes, and the concentrations of seven decreased compounds were the main contributors to PC1, among which EAD, ET-III, ET-IV, ET-VI, ET-VIII, TFs, CTs, ABTS, and FRAP were the most significant; whereas, ET-II, FG, AG, LG-I, and LG-II represented negative loadings in PC1. Then, GA, FO, EA, ET-IX, DPPH, and OPs were the most dominant contributors to PC2. 

The score plots (shown as triangles in Figure 3) manifested that the greatest difference was obtained when the PLI was stored at 0 and 24 h. The PC1 classified the storage times into two groups, namely before-4 h and after-4 h of storage according to the decreased and increased compounds. A classification of values at 0 and 24 h with values at 2–8 h was detected by PC2. Storage at 0 h revealed a tendency to higher antioxidant capacities, higher amounts of phenolic classes, and higher concentrations of seven decreased phenolics, in comparison to the storage at 24 h, which was characterized by the concentrations of 11 increased compounds with the exception of punicalin. However, no clear discrimination during 2–8 h of storage was characterized by any loading value.

## 4. Conclusions

The present study reported the variations of polyphenolic composition and in vitro antioxidant activity of freshly prepared pomegranate (*Punica granatum* L.) leaf infusion (PLI) over one-day storage (0, 2, 4, 6, 8, and 24 h). The results showed that there were no significant changes for the TF and CT contents during 2–8 h storage, meanwhile, the content of TPs and OPs maintained high stability within 24 h of storage. Moreover, our findings illustrated that the PLI exhibited a strong capacity of free radical scavenging and ferric reducing power, which retained over 90% of its capacity after one-day storage. 

Overall, this study demonstrated that PLI over one-day storage possessed good stability of the total polyphenolic contents and antioxidant capacities. This stability was possibly associated with the transformational actions of the polyphenolic profiles where we found seven decreased and eleven increased polyphenols. Consequently, this infusion can serve as a natural antioxidant and green alternative as well as being a stable by-product for the valorization in the food-pharma and nutraceutical industries. Future research may consider its application in preclinical and clinical trials to better exploit its specific therapeutical profiles regarding the health benefits for human beings. 

## Figures and Tables

**Table 1:** Change of phenolic content and antioxidant activity of pomegranate leaf infusion by spectrophotometric assays.

Spectrophotometric Analysis ^1^		Storage Time (Hours) ^2^						Degradation	*p*-Value (2–8 h)	*p*-Value (0–24 h)
		0	2	4	6	8	24			
Phenolic content	Total phenols (mg GA g^−1^ DW)	133.47 ± 3.40 ^a^	132.02 ± 5.01 ^a^	132.49 ± 2.35 ^a^	132.34 ± 0.46 ^a^	129.67 ± 4.50 ^a^	131.40 ± 1.60 ^a^	1.55%	0.43	0.80
	*Ortho*-diphenols (mg GA g^−1^ DW)	244.25 ± 3.59 ^a^	238.63 ± 2.32 ^a^	240.01 ± 2.14 ^a^	237.24 ± 3.25 ^a^	240.42 ± 0.63 ^a^	239.91 ± 3.57 ^a^	1.78%	0.70	0.12
	Flavonoids (mg RU g^−1^ DW)	55.84 ± 0.45 ^a^	54.86 ± 0.73 ^ab^	54.46 ± 0.54 ^abc^	53.67 ± 0.78 ^bc^	53.81 ± 0.22 ^bc^	53.30 ± 0.11 ^c^	4.54%	0.61	0.00
	Tannins (mg EC g^−1^ DW)	124.20 ± 3.67 ^a^	110.60 ± 3.50 ^b^	109.35 ± 1.11 ^bc^	107.37 ± 2.90 ^bc^	105.68 ± 3.42 ^bc^	102.15 ± 1.24 ^c^	17.75%	0.27	0.00
Antioxidant capacity (mmol Trolox g^−1^ DW)	ABTS scavenging	1.80 ± 0.00 ^a^	1.72 ± 0.02 ^b^	1.71 ± 0.01 ^b^	1.69 ± 0.04 ^b^	1.68 ± 0.04 ^b^	1.66 ± 0.03 ^b^	7.93%	0.63	0.00
	DPPH scavenging	1.69 ± 0.01 ^a^	1.63 ± 0.04 ^ab^	1.60 ± 0.03 ^ab^	1.54 ± 0.03 ^b^	1.56 ± 0.03 ^b^	1.59 ± 0.06 ^b^	5.92%	0.14	0.00
	FRAP assay	2.23 ± 0.01 ^a^	2.21 ± 0.01 ^a^	2.09 ± 0.01 ^b^	2.06 ± 0.01 ^bc^	2.03 ± 0.02 ^c^	2.03 ± 0.03 ^c^	9.29%	0.00	0.00

^1^ Abbreviations are as follows: GA, gallic acid; RU, rutin; EC, epicatechin; DW, dry weight; ABTS: 2,2′-azino-bis (3-ethylbenzothiazoline-6-sulfonic acid) diammonium salt; DPPH: 2,2-diphenyl-1-picrylhidrazyl; and FRAP: ferric reducing antioxidant power. ^2^ Values are presented as the mean ± SD (*n* = 3) for each phenolic group and each antioxidant activity assay. Mean values followed by different superscript lowercase letters report significant differences between different storage times, according to Tukey’s multiple range test.

**Table 2:** The polyphenolic profile of pomegranate leaf infusion at 0 h of storage by RP-HPLC-DAD as well as their respective retention times and decrease or increase percentage (%).

Peak NO. ^1^	Retention Time (min)	Identified Compounds ^2^	*Decrease*/Increase Percentage (%)	*p*-Value ^3^
1	10.98	Gallic acid	7.59	***
2	11.12	*Flavan-3-ols*	4.11	***
3	12.73	Punicalin A and B	5.14	*
4	13.38	*Ellagic acid derivatives*	2.48	***
5	15.25	Ellagitannin I	7.37	***
6	16.31	*Flavanone glycoside I*	-	*-*
7	16.54	Ellagitannin II	11.99	***
8	17.24	*Ellagitannin III (galloyl-glucose)*	12.82	***
9	19.01	*Ellagitannin IV (punicalagin A)*	5.29	***
10	20.14	Flavonol glycoside	52.98	***
11	20.37	Ellagitannin V	50.08	***
12	21.30	*Ellagitannin VI*	19.36	***
13	22.56	*Ellagitannin VII (punicalagin B)*	3.93	***
14	23.33	*Ellagitannin VIII*	4.45	***
15	24.35	Ellagic acid	36.39	***
16	24.90	Ellagitannin IX	8.41	***
17	25.10	Flavanone glycoside II	-	-
18	26.44	Apigenin glycoside	31.98	***
19	27.13	Luteolin glycoside I	35.09	***
20	28.38	Luteolin glycoside II	36.03	***

^1^ Peak number and retention time showed according to Figure S1. ^2^ Text in italics represented the compounds in decreasing concentrations. ^3^ Significant differences of polyphenolic compounds among different storage time were denoted as * at *p* < 0.05, and *** at *p* < 0.001, according to Tukey’s multiple range test.

**Table 3:** Pearson correlation coefficient (*r*) between various phenolic compositions and properties.

	GA ^2^	FO	PU	EAD	ET-I	ET-II	ET-III	ET- IV	FG	ET-V	ET- VI	ET- VII	ET- VIII	EA	ET- IX	AG	LG-I	LG- II	ABTS	DPPH	FRAP	TPs	OPs	TFs
FO	0.62																							
PU	−0.06	−0.68																						
EAD	−0.48	0.23	−0.81																					
ET-I	0.50	−0.14	0.60	−0.84 *																				
ET-II	0.30	−0.44	0.76	−0.82 *	0.94 **^,1^																			
ET-III	−0.58	0.20	−0.69	0.89 *	−0.95**	−0.95 **																		
ET-IV	−0.21	0.54	−0.93 **	0.85 *	−0.58	−0.74	0.76																	
FG	0.51	−0.26	0.73	−0.90 *	0.96 **	0.97 **	−0.99 ***	−0.77																
ET-V	0.55	−0.17	0.63	−0.85 *	0.98 ***	0.96 **	−0.99 ***	−0.66	0.99 ***															
ET-VI	−0.55	0.24	−0.72	0.91 *	−0.94 **	−0.95 **	0.99 ***	0.79	−0.99 ***	−0.98 ***														
ET-VII	−0.22	0.49	−0.80	0.73	−0.37	−0.54	0.61	0.95 **	−0.60	−0.47	0.64													
ET-VIII	−0.40	0.40	−0.87 *	0.93 **	−0.69	−0.79	0.85 *	0.97 **	−0.85 *	−0.76	0.87 *	0.92 **												
EA	0.60	0.04	0.40	−0.77	0.95 **	0.82 *	−0.86 *	−0.41	0.87 *	0.91 *	−0.85 *	−0.22	−0.57											
ET-IX	0.78	0.15	0.47	−0.86 *	0.92 **	0.79	−0.93 **	−0.57	0.91 *	0.93 **	−0.92 *	−0.43	−0.72	0.93 **										
AG	0.06	−0.50	0.75	−0.73	0.89 *	0.93 **	−0.80	−0.59	0.85 *	0.85 *	−0.81	−0.34	−0.62	0.78	0.66									
LG-I	0.03	−0.53	0.75	−0.72	0.88 *	0.93 **	−0.79	−0.60	0.84 *	0.84 *	−0.80	−0.35	−0.62	0.76	0.64	0.99 ***								
LG-II	0.06	−0.42	0.69	−0.72	0.88 *	0.88 *	−0.75	−0.51	0.81	0.82 *	−0.76	−0.25	−0.56	0.80	0.66	0.99 ***	0.98 ***							
ABTS	−0.08	0.66	−0.95 **	0.84 *	−0.77	−0.92 *	0.84 *	0.91 *	−0.87 *	−0.81	0.86 *	0.76	0.88 *	−0.58	−0.62	−0.86 *	−0.87 *	−0.79						
DPPH	0.02	0.72	−0.90 *	0.75	−0.44	−0.64	0.60	0.95 **	−0.63	−0.50	0.64	0.94 **	0.89 *	−0.28	−0.37	−0.54	−0.55	−0.46	0.86 *					
FRAP	−0.26	0.52	−0.83 *	0.91 *	−0.75	−0.84 *	0.82 *	0.88 *	−0.85 *	−0.78	0.85 *	0.80	0.92 **	−0.68	−0.69	−0.74	−0.74	−0.71	0.89 *	0.88 *				
TPs	0.03	0.35	−0.82	0.79	−0.61	−0.59	0.54	0.63	−0.60	−0.54	0.57	0.45	0.64	−0.51	−0.51	−0.72	−0.72	−0.77	0.71	0.60	0.66			
OPs	0.21	0.70	−0.77	0.38	−0.19	−0.46	0.39	0.78	−0.40	−0.29	0.42	0.75	0.63	0.10	−0.07	−0.36	−0.37	−0.23	0.72	0.76	0.46	0.33		
TFs	−0.25	0.56	−0.91 *	0.90 *	−0.78	−0.91 *	0.89 *	0.95 **	−0.91 *	−0.83 *	0.91 *	0.84 *	0.96 **	−0.62	−0.70	−0.79	−0.79	−0.71	0.98 ***	0.89 *	0.94 **	0.65	0.68	
CTs	−0.01	0.70	−0.94 **	0.79	−0.76	−0.92 *	0.81 *	0.87 *	−0.85 *	−0.79	0.84 *	0.70	0.84 *	−0.56	−0.57	−0.89 *	−0.89 *	−0.82	0.99 ***	0.83 *	0.86 *	0.70	0.72	0.95 **

^1^ Values in bold represent the * significant at *p* < 0.05; ** significant at *p* < 0.01; and *** significant at *p* < 0.001. ^2^ Abbreviations: GA, gallic acid; FO, flavan-3-ols; PU, punicalins; EAD, ellagic acid derivative; ET-I, ellagitannin I; ET-II, ellagitannin II; ET-III, ellagitannin III; ET-IV, ellagitannin IV; FG, flavonol glycoside; ET-V, ellagitannin V; ET-VI, ellagitannin VI; ET-VII, ellagitannin VII; ET-VIII, ellagitannin VIII; EA, ellagic acid; ET-IX, ellagitannin IX; AG, apigenin glycoside; LG-I, luteolin glycoside I; LG-II, luteolin glycoside II; TPs, total phenols; OPs, *ortho*-diphenols; TFs, total flavonoids, and CTs, condensed tannins.

## Data Availability

All data are contained in this article and Supplementary Materials.

## Supplementary Materials

The following are the supplementary data to this article at https://www.mdpi.com/article/10.3390/antiox10081187/s1. Table S1. Identified compounds associated with the peaks of Figure S1. Table S2. Factor loadings for illustrating the interpretation of Figure 3; Figure S1. Representative RP-HPLC-DAD chromatogram (at 280 nm) of pomegranate leaf infusion during 0–24 h storage at room temperature.
