# Supplementary material for: Variation of the Polyphenolic Composition and Antioxidant Capacity of Freshly Prepared Pomegranate Leaf Infusions over One-Day Storage
Source: Antioxidants (Basel). 2021 Jul 26;10(8):1187. doi: 10.3390/antiox10081187 (PMC8388870; doi:10.3390/antiox10081187)
Supplement: Supplementary file 1 [file antioxidants-10-01187-s001.zip › antioxidants-1280972-Supple new.pdf]

Variation of polyphenolic composition and antioxidant capacity of freshly prepared pomegranate leaf infusions over one-day storage

Manyou Yu [a]*, Irene Gouvinhas [a], Ana Barros [a, b]

[a] Centre for the Research and Technology of Agro-Environmental and Biological Sciences (CITAB) / Inov4Agro (Institute for Innovation, Capacity Building and Sustainability of Agri-Food Production), University of Trás-os-Montes and Alto Douro (UTAD), 5000-801, Vila Real, Portugal. igouvinhas@utad.pt (Irene Gouvinhas)

[b] Department of Chemistry, School of Life Sciences and Environment, UTAD, Quinta de Prados; 5000-801, Vila Real, Portugal. abarros@utad.pt. (Ana Barros)

***** Correspondence: jiangymy518@163.com. (Manyou Yu)

Captions

Table S1. Identified compounds associated with the peaks of Figure S1.

Table S2. Factor loadings for illustrating the interpretation of Figure 3.

Figure S1. Representative RP-HPLC-DAD chromatogram (at 280 nm) of pomegranate leaf infusion during 0–24 h storage at room temperature.

Table S1. Identified compounds associated with the peaks of Figure S1.

Peak No.	Identified Compounds	Relevant References
1	Gallic acid	Li, *et al.* [1]; Li, *et al.* [2]; Swilam and Nematallah [3];
2	Flavan-3-ols	de Oliveira, *et al.* [4]; Lansky and Newman [5];
3	Punicalin (A+B)	Pinheiro, *et al.* [6]; Tanaka, *et al.* [7]; Li, *et al.* [2]
4	Ellagic acid derivative	Pinheiro, *et al.* [6]; Marques, *et al.* [8]; Acquadro, *et al.* [9]
5	Ellagitannin I	Pinheiro, *et al.* [6]
6	Flavanone glycoside I	Heber, *et al.* [10]; Srivastava, *et al.* [11]
7	Ellagitannin II	Gil, *et al.* [12]
8	Ellagitannin III (galloyl-glucose)	Gil, *et al.* [12]; Pinheiro, *et al.* [6]; Nawwar, *et al.* [13]; Acquadro, *et al.* [9]; Swilam and Nematallah [3];
9	Ellagitannin IV (punicalagin A)	Gil, *et al.* [12]; Çam and Hışıl [14]; Russo, *et al.* [15]; Swilam and Nematallah [3]
10	Flavonol glycoside	Pinheiro, *et al.* [6]; Marques, *et al.* [8]; de Oliveira, *et al.* [4]
11	Ellagitannin V	Pinheiro, *et al.* [6]
12	Ellagitannin VI	Russo, *et al.* [15]
13	Ellagitannin VII (punicalagin B)	Çam and Hışıl [14]; Fischer, *et al.* [16]; Russo, *et al.* [15]
14	Ellagitannin VIII	Fischer, *et al.* [16]
15	Ellagic acid	Wang, *et al.* [17]; Li, *et al.* [1]; Çam and Hışıl [14]; Li, *et al.* [2]; Acquadro, *et al.* [9]
16	Ellagitannin IX	Fischer, *et al.* [16]
17	Flavanone glycoside II	Heber, *et al.* [10]; Srivastava, *et al.* [11];
18	Apigenin glycoside	Nawwar, *et al.* [18]; Acquadro, *et al.* [9]
19	Luteolin glycoside I	Nawwar, *et al.* [18]; Acquadro, *et al.* [9]
20	Luteolin glycoside II	Nawwar, *et al.* [18]; Acquadro, *et al.* [9]

Table S2. Factor loadings for illustrating the interpretation of Figure 3.

Factor No.	Factors	PC 1	PC 2
1	Gallic acid	-0.011	0.034
2	Flavan-3-ols	0.024	0.016
3	Punicalin (A+B)	-0.030	0.005
4	Ellagic acid derivative	0.011	-0.010
5	Ellagitannin I	-0.011	0.031
6	Ellagitannin II	-0.039	0.073
7	Ellagitannin III (galloyl-glucose)	0.081	-0.132
8	Ellagitannin IV (punicalagin A)	0.635	0.115
9	Flavonol glycoside	-0.075	0.127
10	Ellagitannin V	-0.260	0.596
11	Ellagitannin VI	0.139	-0.208
12	Ellagitannin VII (punicalagin B)	0.580	0.497
13	Ellagitannin VIII	0.079	-0.010
14	Ellagic acid	-0.098	0.402
15	Ellagitannin IX	-0.017	0.041
16	Apigenin glycoside	-0.073	0.195
17	Luteolin glycoside I	-0.013	0.034
18	Luteolin glycoside II	-0.006	0.018
19	ABTS scavenging activity	0.002	-0.002
20	DPPH scavenging activity	0.003	0.001
21	FRAP ferric reducing power	0.004	-0.002
22	Total phenols	0.041	-0.044
23	*Ortho*-diphenols	0.093	0.094
24	Total flavonoids	0.047	-0.026
25	Condensed tannins	0.352	-0.294

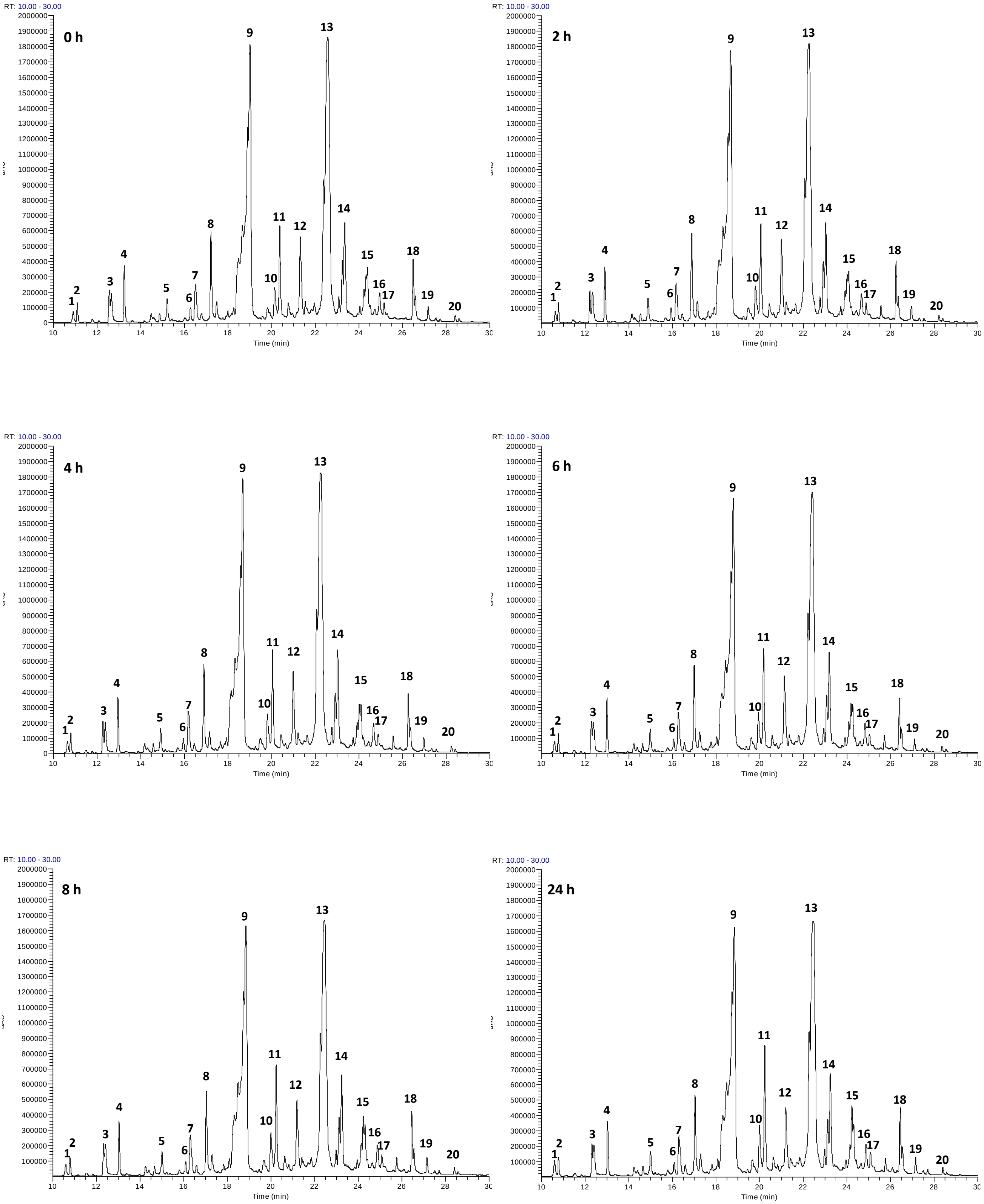

Figure S1. Representative RP-HPLC-DAD chromatogram (at 280 nm) of pomegranate leaf infusion during 0–24 h storage at room temperature.

References:

1. Li, J.; Wang, G.; Hou, C.; Li, J.; Luo, Y.; Li, B. Punicalagin and ellagic acid from pomegranate peel induce apoptosis and inhibits proliferation in human HepG2 hepatoma cells through targeting mitochondria. *Food Agric. Immunol.* **2019**, *30*, 897-912, doi:10.1080/09540105.2019.1642857.
2. Li, R.; Chen, X.G.; Jia, K.; Liu, Z.P.; Peng, H.Y. A systematic determination of polyphenols constituents and cytotoxic ability in fruit parts of pomegranates derived from five Chinese cultivars. *SpringerPlus* **2016**, *5*, 914, doi:10.1186/s40064-016-2639-x.
3. Swilam, N.; Nematallah, K.A. Polyphenols profile of pomegranate leaves and their role in green synthesis of silver nanoparticles. *Sci. Rep.* **2020**, *10*, 14851, doi:10.1038/s41598-020-71847-5.
4. de Oliveira, J.F.F.; Garreto, D.V.; da Silva, M.C.P.; Fortes, T.S.; de Oliveira, R.B.; Nascimento, F.R.F.; Da Costa, F.B.; Grisotto, M.A.G.; Nicolete, R. Therapeutic potential of biodegradable microparticles containing *Punica granatum* L. (pomegranate) in murine model of asthma. *Inflamm. Res.* **2013**, *62*, 971-980, doi:10.1007/s00011-013-0659-3.
5. Lansky, E.P.; Newman, R.A. *Punica granatum* (pomegranate) and its potential for prevention and treatment of inflammation and cancer. *J. Ethnopharmacol.* **2007**, *109*, 177-206, doi:10.1016/j.jep.2006.09.006.
6. Pinheiro, A.J.M.C.R.; Gonçalves, J.S.; Dourado, Á.W.A.; de Sousa, E.M.; Brito, N.M.; Silva, L.K.; Batista, M.C.A.; de Sá, J.C.; Monteiro, C.R.A.V.; Fernandes, E.S.; et al. *Punica granatum* L. Leaf Extract Attenuates Lung Inflammation in Mice with Acute Lung Injury. *J. Immunol. Res.* **2018**, *2018*, 6879183, doi:10.1155/2018/6879183.
7. Tanaka, T.; Nonaka, G.-I.; Nishioka, I. Tannins and Related Compounds. XL.: Revision of the Structures of Punicalin and Punicalagin, and Isolation and Characterization of 2-O-Galloylpunicalin from the Bark of *Punica granatum* L. *Chem. Pharm. Bull.* **1986**, *34*, 650-655, doi:10.1248/cpb.34.650.
8. Marques, L.C.; Pinheiro, A.J.; Araújo, J.G.; de Oliveira, R.A.; Silva, S.N.; Abreu, I.C.; de Sousa, E.M.; Fernandes, E.S.; Luchessi, A.D.; Silbiger, V.N. Anti-inflammatory effects of a pomegranate leaf extract in LPS-induced peritonitis. *Planta Med.* **2016**, *82*, 1463-1467, doi:10.1055/s-0042-108856.
9. Acquadro, S.; Civra, A.; Cagliero, C.; Marengo, A.; Rittà, M.; Francese, R.; Sanna, C.; Bertea, C.; Sgorbini, B.; Lembo, D. *Punica granatum* Leaf Ethanolic Extract and Ellagic Acid as Inhibitors of Zika Virus Infection. *Planta Med.* **2020**, *86*, 1363-1374, doi:10.1055/a-1232-5705.
10. Heber, D.; Schulman, R.N.; Seeram, N.P. *Pomegranates: ancient roots to modern medicine*, 1st ed.; Taylor & Francis CRC press: Boca Raton, FL, USA, 2006.
11. Srivastava, R.; Chauhan, D.; Chauhan, J. A flavonoid diglycoside from *Punica granatum*. Pharm. Biol. **2001**, *39 (2)*: 155-157, doi:10.1076/phbi.39.2.155.6254.
12. Gil, M.I.; Tomás-Barberán, F.A.; Hess-Pierce, B.; Holcroft, D.M.; Kader, A.A. Antioxidant Activity of Pomegranate Juice and Its Relationship with Phenolic Composition and Processing. *J. Agric. Food Chem.* **2000**, *48*, 4581-4589, doi:10.1021/jf000404a.
13. Nawwar, M.A.M.; Hussein, S.A.M.; Merfort, I. NMR spectral analysis of polyphenols from *Punica granatum*. *Phytochemistry* **1994**, *36*, 793-798, doi:10.1016/S0031-9422(00)898 20-9.
14. Çam, M.; Hışıl, Y. Pressurised water extraction of polyphenols from pomegranate peels. *Food Chem.* **2010**, *123*, 878-885, doi:10.1016/j.foodchem.2010.05.011.

15. Russo, M.; Fanali, C.; Tripodo, G.; Dugo, P.; Muleo, R.; Dugo, L.; De Gara, L.; Mondello, L. Analysis of phenolic compounds in different parts of pomegranate (*Punica granatum*) fruit by HPLC-PDA-ESI/MS and evaluation of their antioxidant activity: application to different Italian varieties. *Anal. Bioanal. Chem.* **2018**, *410*, 3507-3520, doi:10.1007/s00216-018-0854-8.
16. Fischer, U.A.; Carle, R.; Kammerer, D.R. Identification and quantification of phenolic compounds from pomegranate (*Punica granatum* L.) peel, mesocarp, aril and differently produced juices by HPLC-DAD–ESI/MSn. *Food Chem.* **2011**, *127*, 807-821, doi:10.1016/j.foodchem.2010.12.156.
17. Wang, C.; Shi, L.; Fan, L.; Ding, Y.; Zhao, S.; Liu, Y.; Ma, C. Optimization of extraction and enrichment of phenolics from pomegranate (*Punica granatum* L.) leaves. *Ind. Crops Prod.* **2013**, *42*, 587-594, doi:10.1016/j.indcrop.2012.06.031.
18. Nawwar, M.A.M.; Hussein, S.A.M.; Merfort, I. Leaf phenolics of *Punica granatum*. *Phytochemistry* **1994**, *37*,1175-1177, doi:10.1016/S0031-9422(00)89552-7.
